# Genetic basis of resistance in hosts facing alternative infection strategies by a virulent bacterial pathogen

**DOI:** 10.1093/g3journal/jkae302

**Published:** 2024-12-21

**Authors:** Eglantine Mathieu-Bégné, Sabrina Gattis, Dieter Ebert

**Affiliations:** Department of Environmental Sciences, Zoology, University of Basel, CH-4051 Basel, Switzerland; Department of Environmental Sciences, Zoology, University of Basel, CH-4051 Basel, Switzerland; School of Zoology, George S. Wise Faculty of Life Sciences, Tel Aviv University, Tel Aviv 69978, Israel; Department of Environmental Sciences, Zoology, University of Basel, CH-4051 Basel, Switzerland

**Keywords:** coevolution, Mendelian locus, fine-mapping, epistatic interactions, infection strategies

## Abstract

Having alternative infection routes is thought to help parasites circumvent host resistance, provided that these routes are associated with different host resistance loci. This study tests this postulate by examining whether alternate infection routes of the parasite *Pasteuria ramosa* are linked to distinct resistance loci in its crustacean host, *Daphnia magna*. We focus on the *P. ramosa* isolate P15, which can attach and penetrate the host through either the hindgut or the foregut. Using a global panel of 174 *D. magna* genotypes supplemented with breeding experiments, we analyzed resistance patterns for each of these infection routes. Our findings confirm our hypothesis in *D. magna*, hindgut attachment is determined by the D locus, while foregut attachment is controlled by a newly identified G locus. We established a gene model for the G locus that indicated Mendelian segregation and epistatic interaction with at least one other resistance locus for *P. ramosa*, the C locus. Using genomic Pool-sequencing data, we localized the G locus within a known Pasteuria Resistance Complex on chromosome 4 of *D. magna*, whereas the D locus is on chromosome 7. Two candidate genes for the G locus, belonging to the Glycosyltransferase gene family, were identified. Our study sheds new light on host–parasite coevolution and enhances our understanding of how parasites evolve infection strategies.

## Introduction

The process by which a parasite (incl. pathogens) infects a host is often depicted as a stepwise process starting with host encounter, followed by host entry via a specific route, then within-host growth, parasite proliferation and transmission (Fenton *et al*. 2012; Hall *et al*. 2017; Lievens *et al*. 2018; Hite 2020; Izhar *et al*. 2020). In this model, the parasite follows a linear path, where at each step the parasite interacts with different aspects of its host. The host can evolve resistance by blocking the parasite at any of these different steps; the earlier this happens, the less damage the parasite causes (Lievens *et al*. 2018; Izhar *et al*. 2020). However, parasites may evolve ways to circumvent host resistance, leading to coevolution.

Coevolution between host and parasite is based on reciprocal selection and is thought to drive diversity among organisms (Hamilton 1982; Mouritsen and Poulin 2005; Spurgin and Richardson 2010; Bever *et al*. 2015). Knowing the genomic architecture that underlies resistance traits in hosts and virulence traits in pathogens is thus critical to our understanding of host–parasite coevolution (Kurtz *et al*. 2016; Ebert and Fields 2020). It comes as no surprise that identifying the loci of host and parasite interactions has been a persistent focus in disease biology (e.g. Shea *et al*. 1996; Su *et al*. 2002; Beraldi *et al*. 2007; Turner *et al*. 2011; Routtu and Ebert 2015). Research has documented strong and specific genetic associations between hosts and their parasites, for example, between mosquitoes and the blood parasite *Plasmodium falciparum* (Lambrechts *et al*. 2005), between the filter feeding crustacean *Daphnia magna* and its obligate bacteria pathogen *Pasteuria ramosa* (Carius *et al*. 2001), between the blood parasite *Schistosoma* and its intermediate host the aquatic snail *Biomphalaria* (Webster and Woolhouse 1998), and in human major histocompatibility complex (MHC) in relation to diverse pathogens (Råberg 2023). Relationships between the host resistance locus and the parasite infectivity locus have also been documented at the gene level and evidences for the gene-for-gene model and the matching allele model have been provided (Dodds *et al*. 2006; Luijckx *et al*. 2013; Råberg 2023). For example, such evidences at the gene level have been documented between human MHC genes and some pathogens (Råberg 2023) and between plant R gene and flax rust virulence genes (Dodds *et al*. 2006), while the genetic models for resistance loci of *D. magna* against *P. ramosa* are supporting the Matching Alleles Model (Luijckx *et al*. 2013).

The stepwise model of infection often neglects the fact that for the parasite, the host constitutes a heterogeneous landscape (Lydecker *et al*. 2019). For ectoparasites, the host's body surface presents several potential microhabitats that are not equivalent; some locations may be preferred as an entry point by a given parasite (Loot *et al*. 2004; Pigeault *et al*. 2020). Endoparasites typically have defined entry points to the host, such as the skin, the lung, the gut epithelium, and the mucosal surfaces of genital organs (for sexually transmitted diseases). This goes down even to specific cell types a parasite can colonize (Iyer *et al*. 2007). Because parasites view hosts as heterogeneous, they have potentially diverse infection routes, and by evolving the ability to use alternative paths to enter the host, they can overcome host resistance. However, alternative infections routes only benefit the parasite if host resistance to one route does not simultaneously block all other routes—i.e. if the resistance mechanism is route-specific. Therefore, when parasites successfully use different host entry routes, we expect host resistance polymorphisms to be specific to each individual route, i.e. different entry points are associated with different host genes. Thus, the host is expected to have different genes associated with resistance to the same parasite line.

**Fig. 1.**
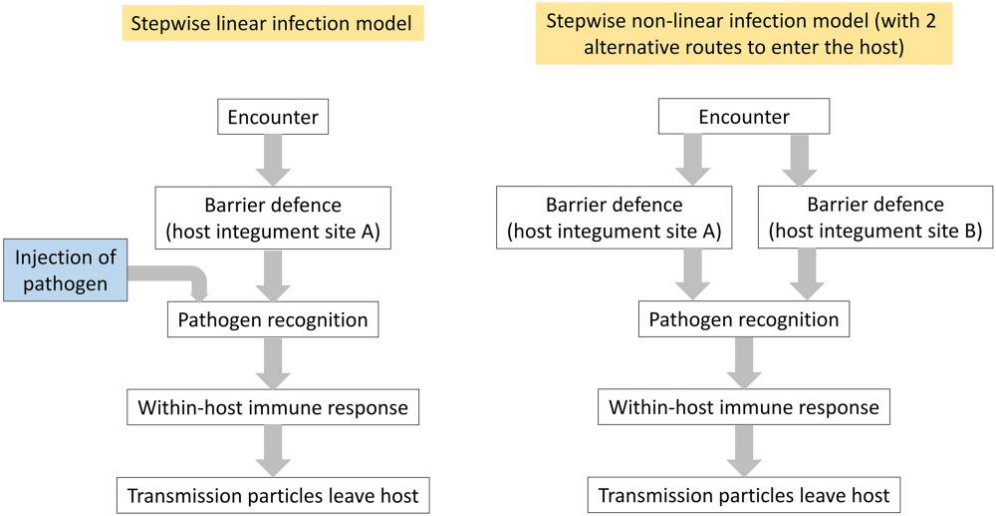
Linear (left) vs nonlinear (right) infection models. The left side text box indicates systemic infections, e.g. by pricking the skin with a needle dipped in a pathogen culture, circumventing the first line of defense in linear infection models. This shortens the model, but does not present an alternative path for the pathogen. The non-linear infection model (right) shows two alternative routes of infection, where the pathogen can enter the host in two different sites.

The use of alternative host entry points suggests that pathogen infection can occur as a non-linear process (with different potential branches) rather than a linear process. The evolution of host resistance and parasite virulence and its genomic basis remains relatively unexplored in the context of non-linear infection models. Part of the reason is because the use of alternative infection paths by parasites is rarely considered explicitly within a non-linear model of infection. For instance, it has been suspected that oral and systemic infections of *Drosophila melanogaster* by *Pseudomonas entomophila* are associated with different resistance genes (see Martins *et al*. 2013); however, here, the different infection routes are actually two different steps of a linear infection model: in one case, the encounter occurs as *in natura* (i.e. oral infection) while in the other case the pathogen circumvents the encounter step to start directly at the step of pathogen recognition by the host (i.e. systemic infection by pricking the cuticle of the host with an pathogen-loaded needle). Hence, there is often confusion in the literature between a “shortened” linear infection model where the encounter is circumvented and a non-linear infection model, where a pathogen naturally infects through a different host entry point (e.g. Eleftherianos *et al*. 2022). Linear and nonlinear infection models are conceptually different (see Fig. 1). Here, we propose that a nonlinear infection model has different evolutionary outcomes from linear infection models, which can lead to host loci specific to a pathogen's particular infection route.

The freshwater crustacean *D. magna* is often infected with the obligate bacterial parasite *P. ramosa*. According to data presented by Bento *et al*. (2020), a particular isolate (P15) of the parasite can enter through the host cuticle via two different natural routes: the esophagus (foregut) and the hindgut. Attachment to the gut cuticle is a strong indicator of successful infection (Duneau *et al*. 2011; Bento *et al*. 2020). Hindgut attachment may happen with a lower likelihood, and thus lower fitness for the parasite because spores need to pass through the entire gut before reaching the attachment site. Previously, resistance loci have been identified for parasites that enter the host through a specific site. For example, *P. ramosa* isolates P15 and P21 entering through the hindgut are associated with host resistance loci D and F (Bento *et al*. 2020; Fredericksen *et al*. 2023), while the A, B, and C loci determine resistance to *P. ramosa* isolates C1 and C19 that enter the host exclusively through the foregut (Bento *et al*. 2017). Because resistance to different infection routes has only been characterized so far for different *P. ramosa* isolates it has not been possible to distinguish whether the host resistance loci were an effect of the *P. ramosa* line or of the infection route. More recently, Fredericksen *et al*. (2021) have discovered the potential use of multiple infection routes in about two-thirds of *P. ramosa* isolates, often with different parasite isolates using different combinations of routes (out of 5). This plurality of infection routes within *P. ramosa* lines indicates that a linear infection model does not capture the complexity of the coevolutionary interactions between the host and the parasite. Instead, it is necessary to determine whether parasites using different infection routes interact with the same or with different host genes.

In this study, we test—at both the phenotypic level and the genomic level—the hypothesis that different infection routes used by a single *P. ramosa* isolate are associated with different *D. magna* resistance loci. At the phenotypic level, we characterized the different infection routes (hindgut and foregut) of the *P. ramosa* isolate P15 on a worldwide panel of 174 *D. magna* genotypes. We specifically tested phenotypic associations between attachments made via different routes of infection and between three different *P. ramosa* isolates (our focal isolate P15, and two other isolates (C1 and C19) routinely used in our lab that have a known relationship to a locus often involved in epistatic interactions with other resistance loci (Metzger *et al*. 2016; Ameline *et al*. 2021)). We further conducted selfing experiments on a restricted panel of *D. magna* genotypes to investigate segregation patterns between P15 hindgut and foregut attachments. If different infection routes are indeed associated with different, unlinked host loci, we expect to see that P15 foregut and hindgut attachments are independent of each other. Furthermore, using a pool-sequencing approach, we localized the host loci responsible for P15 foregut attachment within *D. magna* reference genome. The locus connected with P15 hindgut attachment (D locus, on chromosome 7 (Bento *et al*. 2020)) was already known. If our hypothesis is correct, we expect the locus associated with P15 foregut attachment to be in a different position. With this study, we challenge the idea that parasitic infections follow a linear, stepwise process. This is of prime interest, since parasites’ ability to escape host resistance mechanisms can raise health concern as new strategies of infection arise.

## Material and methods

### Attachment patterns investigation on a worldwide panel

To test whether different routes of infection are associated with different host resistance polymorphisms, we first aimed to test co-occurrences in the attachment patterns (i.e. routes of infection) of various *P. ramosa* isolates, including the P15 isolate. We relied on an attachment assessment of *P. ramosa* isolates P15, C1, and C19 on a worldwide panel of 174 *D. magna* genotypes that was published by Bento *et al*. (2020). The dataset includes attachment scores (as binary scores) for each infection route used by each *P. ramosa* isolate on each *D. magna* genotype. We used the R package *cooccur* (Griffith *et al*. 2016) to test for positive and negative associations between attachments of C1, C19, P15 foregut (P15F), and P15 hindgut (P15H). *P*-values of the co-occurrence test were corrected for multiple comparison using Benjamini & Hochberg false discovery rate (Benjamini and Hochberg 1995). Results were presented as a co-occurrence matrix with effect size of the co-occurrence tests and levels of significance. Other metrics used and generated by the test, such as observed and expected occurrences, probability of co-occurrence and corrected *P*-values, are provided in Supplementary Table 1.

### Segregation patterns investigation

We aimed to further refine our understanding of the segregation patterns between *D. magna* resistance loci and specifically between potential loci associated with P15F and P15H attachments, respectively. Breeding experiments were conducted on a representative subset of 39 *D. magna* genotypes (see Supplementary Table 2 for a list). These genotypes were kept under conditions that promoted offspring production by selfing (Ebert 2022; Santos and Ebert 2023). Specifically, selfed F1 offspring were produced by allowing females to mate with their asexually produced sons or brothers. Attachments of C1, C19, and P15 were tested with the attachment test (Duneau *et al*. 2011).

Genotypes were first determined based on the genetic model for C1 and C19 resistance (Bento *et al*. 2017; Ameline *et al*. 2021). Next, genotypes for the locus responsible for P15 foregut attachment were inferred for both parents and selfed offspring. This was done on the basis of hypothesized gene models deduced from previous analyses of resistance loci segregation. We compared phenotype frequencies as expected based on the hypothesized gene models to the observed phenotype frequencies. Recombination rates were assumed to be 0.5.

### Pool-sequencing

We relied on a genomic approach to localize the locus associated with P15F attachment within the *D. magna* genome and to confirm that it differs from the locus associated with P15H attachment. We conducted pool-sequencing with selfed offspring from a genotype that was predicted to be heterozygote for the locus responsible for P15F attachment but homozygote for other known resistance loci (Genotype TN-RA-21 from Tunesia, see Table 1), TN-RA-21 was propagated and allowed to produce resting eggs (sexual eggs = ephippia) by selfing (Santos and Ebert 2023). Resting stages were diapaused and hatched following the method of Santos and Ebert (2023). Each hatchling (F1) was cloned; then six individuals per genotype were exposed to P15 spores to test for P15 foregut attachment (Duneau *et al*. 2011). If over 50% of the individuals showed P15 foregut attachments, they were defined as susceptible; otherwise, they were labeled resistant. Overall, 23 F1 TN-RA-21 were produced and scored as resistant, and 33 F1 TN-RA-21 were produced and scored as susceptible.

**Table 1.** Parental genotype and selfed-offspring resistance phenotypes (i.e. resistotypes) for the four *D. magna* genotypes among the selfed offspring that segregated for *P. ramosa* P15F attachment

Parent genotype ID	Resistotype		Hypothesized genotype (CG loci, model 1 or model 2)	Selfed offspring (F1) resistotype		Nobs	%obs	%exp (model 1 or model 2)	Hypothetized genotype (CG loci, model 1 or model 2)
	C1, C19	P15F		C1, C19	P15F				
CY-PA3-2	RR	S	Ccgg or CcGG	RR	S	10	71.43	75	C-gg or C-GG
				SR	R	4	28.57	25	ccgg or ccGG
ES-HT-1	RR	S	Ccgg or CcGG	RR	S	24	63.16	75	C-gg or C-GG
				SR	R	14	36.84	25	ccgg or ccGG
TN-RA-02	RR	R	CCGg	RR	R	27	87.1	75 or 25	CCG- or CCgg
				RR	S	4	12.9	25 or 75	CCgg or CCG-
TN-RA-21	RR	R	CCGg	RR	R	22	81.48	75 or 25	CCG- or CCgg
				RR	S	5	18.52	25 or 75	CCgg or CCG-

Hypothesized genotypes are given according to genetic model 1 and 2 for the putative G locus. Finally, the percentage of the observed and expected P15 foregut attachments based on the gene model 1 or gene model 2 for the G locus are provided. For the full table of the 39 genotypes used in the breeding experiment, refer to the Supplementary Table 4.

#### DNA extraction and pool-sequencing

Prior to DNA extraction, *Daphnia* were prepared following the protocol described in Dexter *et al*. (2023). Three replicates of resistant and susceptible pools were produced. In order to keep the DNA contribution of each genotype balanced in the pools, two individuals per genotype were added to the pools as described in Supplementary Table 3. DNA was extracted using the Qiagen GenePure DNA Isolation kit and following the DNA-extraction of *Daphnia* and symbionts protocol (dx.doi.org/10.17504/protocols.io.5jyl82n96l2w/v1). Briefly, whole *Daphnia* were denatured using a pestle in lysis solution. Proteinase K was then added to degrade host cuticle, and samples were first incubated overnight at 55°C and then again for half an hour at 37°C with RNase. They were then treated with a protein precipitation solution, and centrifuged to remove precipitates. The supernatant was treated with isopropanol and glycogen as a DNA carrier. The DNA pellet was then washed with 70% ethanol and resuspended in hydration solution prior to DNA quantification. Libraries were prepared and sequenced as paired-end short reads (150 bp) at the Quantitative Genomics Facility service platform Basel (D-BSSE, ETH), Switzerland, using Illumina technology on a novaseq6000 sequencer. The average coverage was 278X and 262X for the resistant and susceptible pools, respectively.

#### Bioinformatic analysis

Paired-end raw reads were quality filtered and trimmed using trimmomatic (Version 0.39). Reads were subsequently mapped onto the *D. magna* reference genome (version 3.1, Cornetti *et al*. 2024) using the mapper BWA (function bwa-mem2 for speed efficiency (Li 2013; Vasimuddin *et al*. 2019)) with the quality filter -*q* set at 20, which allowed for the removal of ambiguously mapped reads. Bam files were sorted by coordinates and indexed. We used the software *Popoolation2* (Kofler *et al*. 2011) to compute allele frequencies and genomic differentiation between resistant and susceptible pools. Specifically, we created a synchronized file for resistant and susceptible pools using SAMtools mpileup function followed by the executable mpileup2sync.jar in *Popoolation2.* This synchronized file contains the allele count for all bases in the reference genome and for all pools being analyzed. We then computed allele frequencies using a minimum count of six, a minimum coverage of 20, a maximum coverage of 200 and set the pool size according to the pool size of each F1 genotypes (i.e. TN-RA-21 F1 resistant = 23, TN-RA-21 F1 susceptible = 33). We further tested for significant differences in allele frequencies using a Fisher Exact Test. The same analyses were run for each replicate of the resistant and susceptible pool to assess results reproducibility.

We tested for allele frequency differences using Fisher Exact Test first, because it is a very common metric for differentiation when working with pool-sequencing data. However, this metric resulted in a very broad peak for the location of the locus along the *D. magna* genome which was informative only at the contig level (i.e. several MBs length). As a clear peak of allelic frequency change was observed consistently in all three replicates (Supplementary Fig. 1), we present results only from the first replicate for subsequent analyses.

To refine the position of the locus, we then used the gene models hypothesized for the locus responsible for P15F attachment. Hypothesized models were based on 1 locus with 2 alleles and perfect dominance. Because this type of model often applies to sex-determining loci, we used the PSASS (Pooled Sequencing Analyses for Sex Signal) tool that is usually used to localize sex determining loci, https://github.com/SexGenomicsToolkit/PSASS (Feron 2020). Using sliding windows across the region of interest, we visualized common differentiation statistics (Fst) and other metrics such as private alleles content in each pool (that is derived from alleles frequencies in each pool) and coverage. Using private alleles content and coverage in each pool, it is possible to refine the position of a locus for which the expected frequencies in the two pools that are contrasted are known, which in our case was provided by the gene models. In order to match Mendelian expected frequencies, we defined heterozygote frequency between 0.30 and 0.70 and homozygote frequencies as superior to 0.90. The results were visualized using modified versions of the functions present in the associate PSASS R package (psass-vis) so as to make them compatible with our own data. We further directly filtered SNPs based on expected major and minor allele frequencies in each pool and extracted from Popoolation2 results to confirm the results provided by PSASS.

#### Candidate genes investigation

To identify potential candidate genes for the host locus responsible for P15 foregut attachment, we first filtered SNPs based on allele frequencies expected in each pool as described above. We selected the most significant SNPs that showed a change in allele frequency between the resistant and the susceptible pool based on the corrected *P*-value (Benjamini—Hochberg correction) of the Fisher Exact Test at a threshold of 1%. These SNPs were then mapped onto the *D. magna* reference genome, and gene model and their position visualized using the Integrative Genome Viewer (Robinson 2011). When a SNP fell onto a gene, the annotation of the gene was further checked for more up-to-date annotations using a blastx query on the NCBI online tool and with the experimental clustered non-redundant database (Sayers *et al*. 2022). SNPs were categorized as falling into a non-coding region or into a coding region. When falling into a coding region, SNPs were further categorized as synonymous or non-synonymous. Note that to do so, we assumed that beside the SNPs that differed between the two pools, the rest of the sequence remained unchanged compared to the reference. As expected, the resistant haplotypes were identical to the sequences found in the reference, which is a genotype that is also resistant to P15F attachment.

## Results and discussion

### Characterization of the routes of infection of P15

**Fig. 2.**
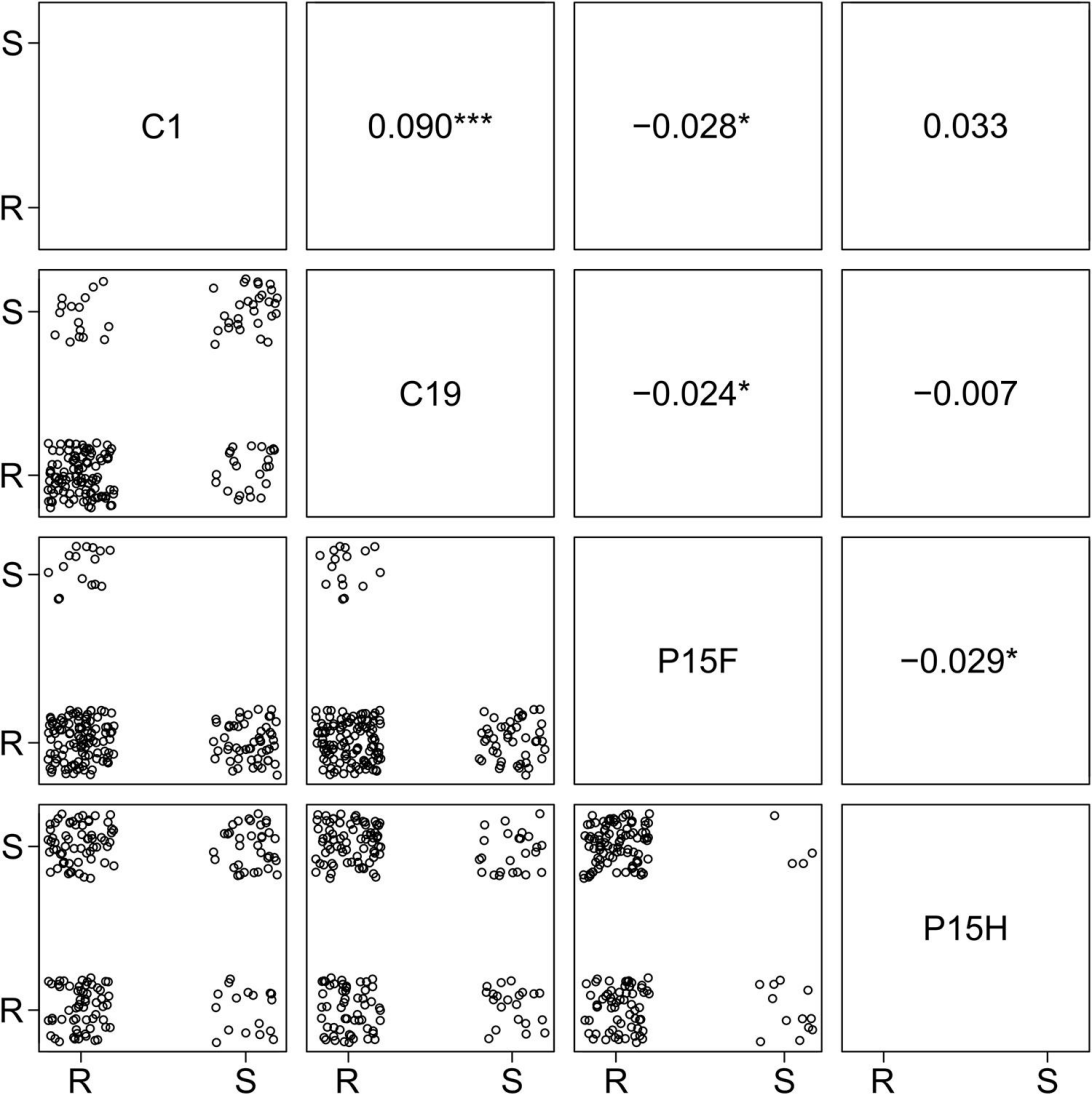
Co-occurrences between the attachments of C1, C19, P15 hindgut (P15H) and P15 foregut (P15F). Circles indicate a *D. magna genotype* as either resistant (R) or susceptible (S) to each *P. ramosa* isolate. Numbers on the upper part of the plot are standardized effect size from the co-occurrence test (i.e. the differences between expected and observed frequency of co-occurrence). Positive effect size indicates a positive co-occurrence, and negative, a negative co-occurrence. Stars indicate the test's level of significance, if any (*** is <0.001, ** is <0.01 and * is <0.05). Raw and corrected p-values are provided in Supplementary Table 1.

Using data from Bento *et al*. (2020) on foregut and hindgut attachments of the P15, C1 and C19 *P. ramosa* isolates on 174 *D. magna* genotypes, we found polymorphism for all three parasite isolates. However, C1 and C19 showed only foregut attachment, whereas P15 showed foregut (P15F) and hindgut (P15H) attachment to some host genotypes. Attachment by the three isolates was not independent of each other: we found a significant positive co-occurrence between C1 and C19 attachments (see Fig. 2). Conversely, we found significant negative co-occurrence between P15F attachment and the three other resistotypes (C1, C19 and P15H, Fig. 2). The negative co-occurrence between P15H and P15F attachments, and the fact that all four possible resistotype combinations were observed, hints that the loci responsible for P15F attachment and P15H attachment might indeed be different (i.e. the locus D). Remarkably, no susceptibility of P15F and the other foregut-attaching isolates, C1 or C19, was observed (Fig. 2). Such impossible combination of resistance phenotype (here susceptibility to both P15F and C1 or C19) can suggest epistatic interactions between their underlying loci, this was hence investigated further in a later section.

In the breeding experiment aimed at refining our understanding of the segregation of resistance loci and testing the segregation between P15F and P15H attachments, selfing of all 39 genotypes produced viable selfed F1 offspring in 31genotypes (Supplementary Table 4). The reassessment of parents and offspring for P15, C1 and C19 attachment confirmed previously reported resistotypes, with C1 and C19 showing only foregut attachment, while P15 showed foregut and hindgut attachments. Selfed F1 offspring of four parental genotypes segregated for P15F attachment (i.e. TN-RA-2, TN-RA-21, ES-HT-1 and CY-PA3-3, see Table 1 and Supplementary Table 4). While TN-RA-2 and TN-RA-21 were resistant to P15F, ES-HT-1 and CY-PA3-2 were susceptible to P15F. Their C1/C19 resistotypes were RR in all cases. These 4 genotypes showed no segregation for P15 hindgut attachment, even though they differed, with TN-RA-2, TN-RA-21 and ES-HT-1 being P15H resistant (P15H-R), whereas CY-PA3-2 was susceptible (P15H-S) (Supplementary Table 4). This suggests that the D locus in these genotypes is homozygote (dd in case of P15H-R, and DD in case of P15H-S) and confirms that the putative locus for P15 foregut attachment segregates independently of the D locus.

### Role of epistatic interaction in P15F attachment

As mentioned above, we did not find P15F susceptibility together with C1 or C19 susceptibility (Fig. 2). The absence of *D. magna* genotypes susceptible to C1, C19 and P15F suggests a possible epistatic interaction between the loci responsible for C1 and C19 resistance and the locus responsible for P15F resistance. The loci responsible for C1 and C19 susceptibility are the A, B and C loci, located within a cluster on chromosome 4 (known as the Pasteuria Resistance Complex, PRC, earlier referred to as the ABC supergene (Bento *et al*. 2017; Fredericksen *et al*. 2023)).

Combining C1 and C19 resistotypes into double types (RR, SS, SR and RS), we observe that SS, SR and RS never coincide with P15F-S, while RR does. Bento *et al*. (2017) showed that the C locus segregates exactly in this way with the phenotypes, such that RR resistotypes are *CC* or *Cc* (C allele is dominant), while SS, RS and SR resistotypes are always *cc*. This observed pattern suggests that the C locus genotype *cc* is resistant to P15F. Since the C locus does not otherwise influence P15F resistance (*CC* and *Cc* genotypes can be P15F-S or P15F-R), we conclude that another locus must influence P15F resistance, but that it acts epistatically with the C locus. We hypothesize that the *cc* genotype nullifies the effect of the P15F resistance locus, regardless of the genotype at P15F resistance locus.

Note that genotypes susceptible to C1 or C19 and P15F at the same time were never observed in Bento *et al*. (2020) data that we are analyzing here. This was also almost unanimously the case for any genotype tested routinely in our lab, hence largely confirming the epistatic interaction between the C and the G locus. However, it is worth noting that few exceptions representing a bit less than 1% of the genotype tested in our lab (i.e. three genotypes) were observed where some susceptible genotypes to P15F were also susceptible to C1 (but not to C19). This very rare exceptions may be explained by a epistatic interaction involving the B locus that appear rare due to the silencing effect of the C and the A locus on the B locus (see Bento *et al*. 2017 for details on the model). However, investigating the potential epistatic role of the B locus in P15F attachment would require a dedicated dataset to be fully explored. In any case, these rare exceptions do not invalidate the hypothesis of an epistatic interaction between the C and the G locus.

Among the 39 *D. magna* genotypes used in the breeding experiment, we also confirmed that P15F attachments were seen only among genotypes with RR resistotypes at C1/C19 (12 of 27 RR genotypes), while genotypes with C1/C19 resistotypes RS, SR, and SS (genotype *cc*) were always resistant to P15F attachment (12 of 39 genotypes, Supplementary Table 4).

Overall, these results suggest that the genetic architecture of P15F attachment is influenced by two loci, with the C locus epistatically masking the effect of the other locus when an individual carries the *cc* genotype. We will hereafter refer to the new locus associated with P15F attachment as the G locus (following the naming sequence that labeled the last discovered locus the F locus).

### Segregation patterns and gene model for P15F resistance locus

We relied on the breeding experiments to further investigate segregation patterns and potential dominance of the locus responsible for P15F attachment. As described above, four genotypes showed segregation for P15 foregut attachment in their selfed offspring. The proportions of susceptible and resistant self F1 offspring to P15F attachment were close to Mendelian. This suggests that a single locus with two alleles and complete dominance could be responsible for P15F resistance (Table 1). Mendelian segregation is commonly observed on the currently-known resistance loci of *D. magna* to *P. ramosa* (Bento *et al*. 2017, 2020; Ameline *et al*. 2021; Fredericksen *et al*. 2023). Two parental genotypes that segregated for P15 foregut attachment in their selfed offspring were susceptible (CY-PA3-2 and ES-HT-1, Table 1) while two others were resistant (TN-RA-2 and TN-RA-21 genotypes, Table 1). The epistatic interaction between the C locus and the G locus deduced above could explain this pattern. The segregation for P15 foregut attachment in CY-PA3-2 and ES-HT-1 is compatible with the masking effect of the C locus on the G locus. Thus, P15F segregation is observed because of segregation of a heterozygote *Cc* parental genotype, while the G locus is homozygote. The TN-RA-21 and TN-RA-2 genotypes showed no segregation at the C locus, with parents and all offspring being resistotype RR (Table 1). This suggests that these two genotypes are homozygote for the dominant allele of the C locus (genotype *CC*) and heterozygote for the G locus. The G locus would therefore be segregating in the offspring of TN-RA-21 and TN-RA-2 (genotype Gg) (Table 1). While the ratios between resistant and susceptible offspring in TN-RA-21 and TN-RA-2 suggest that the locus responsible for P15F attachment is dominant for resistance, the overall low number of offspring segregating for P15F attachment and the strong selection against inbreeding (all selfed F1 are 50% inbreed! ) suggest we take this hypothesis with caution.

**Fig. 3.**
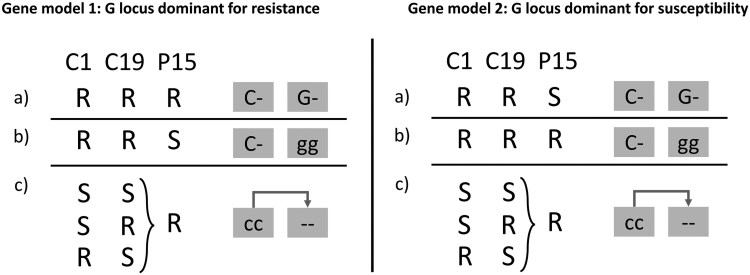
Two alternative genetic models for the G locus. Resistance to *P. ramosa* P15 foregut attachment to be determined by the G locus and its interaction with the C locus. Genotypes at these loci are represented by boxes with grey background. Each locus has two alleles, with upper case denoting dominance. The dash (−) indicates a wildcard, i.e. that the alleles on these positions do not play a role in determining the host's resistotype. a) P15 foregut resistance is dominant (left, model 1) or susceptibility is dominant (right, model 2); b) P15 foregut susceptibility (model 1) or resistance (model 2) is conferred by the recessive alleles on the G locus. c) Epistatic interaction (dark grey arrow): C locus genotype *cc*, nullifies the effect of the G locus, such that homozygosity of the recessive alleles on the C locus masks the expression of alleles on the G locus, making the host resistant to P15 foregut attachment (true for both model 1 and model 2). The letter R and S stand for resistance and susceptibility to the *P. ramosa* isolates listed in the top row.

Two alternative models for the G locus and a summary of the above deductions are presented in Fig. 3. Hypothesized genotypes are reported in Table 1. The G locus is predicted to be a two allele Mendelian locus with total dominance (like other *D. magna* resistance loci against *P. ramosa*). The models differ only in that resistance is dominant in model 1 while susceptibility is dominant in model 2. In both model 1 and 2, the genotypes TN-RA-2 and TN-RA-21 are predicted to be heterozygotes for the G locus. Likewise, epistatic interactions with the C locus are present, such that *cc* genotypes nullify the effect of the G locus. Note, the C locus has previously been shown to interact epistatically with other loci. The dominant C allele nullifies the A and the B loci, while the genotype *cc* nullifies the E locus (Bento *et al*. 2017; Ameline *et al*. 2021). The pivotal role of the epistatic interaction involving the C locus in determining P15F attachment is hence very much in line with observations about other resistance loci against *P. ramosa* in *D. magna*.

### Genomic mapping of the G locus

**Fig. 4.**
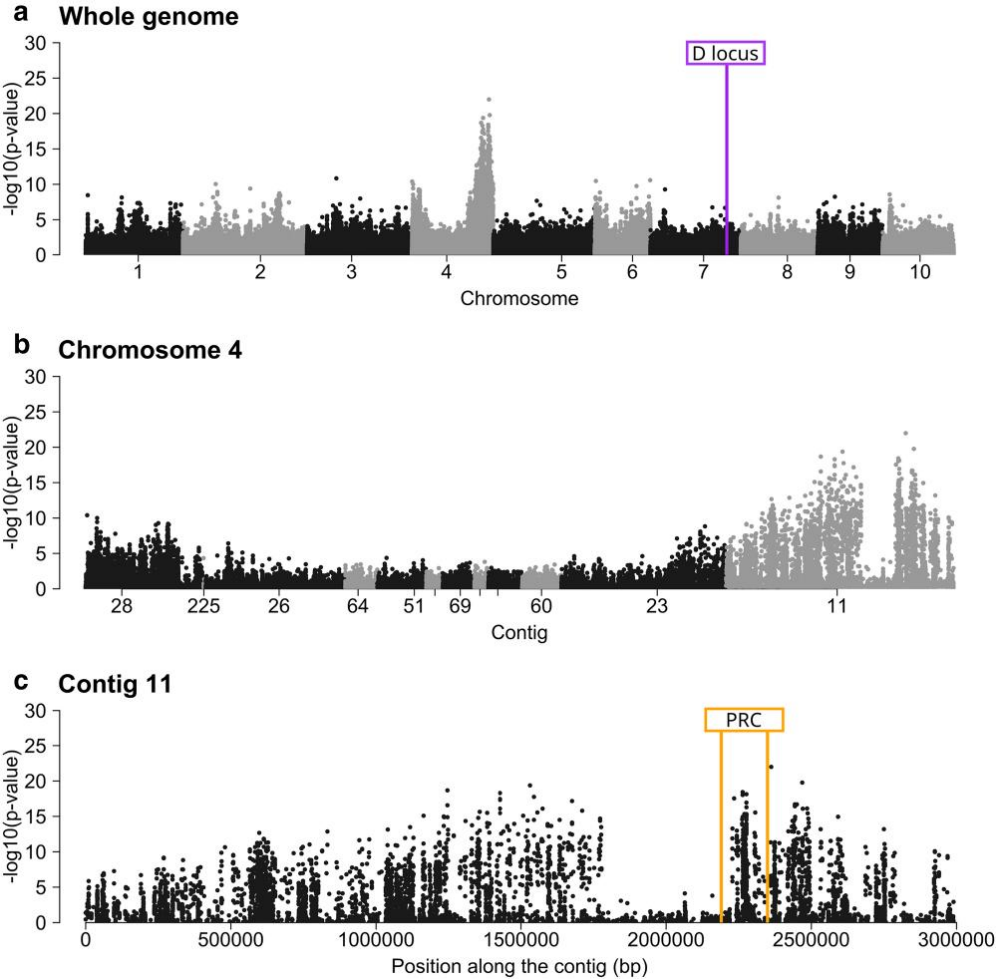
Manhattan plots representing the -log10(*P*-values) of the Fisher Exact Tests conducted on alleles frequencies of the resistant and susceptible pools regarding P15F attachment (replicate 1, see Supplementary Fig. 1 for all three replicates) as a function of genomic positions across the whole genome (a), zoomed in to chromosome 4 (b) and to contig 11 on chromosome 4 (c). The position of the D locus and the A, B, C, F loci cluster (i.e. Pasteuria Ramosa Complex, PRC) are also indicated.

To locate the G locus, we relied on pool sequencing of resistant and susceptible selfed offspring of genotype TN-RA-21 (i.e. predicted heterozygote at the G locus, Table 1). Specifically, we used Fisher Exact Tests to detect significant changes in allele frequencies between a pool of 33 genotypes susceptible to P15F attachment and 23 genotypes resistant to P15F attachment. A clear peak of allelic frequency change was observed on contig 11 of chromosome 4 (Fig. 4), indicating that the G locus is definitely in a different region than the D locus (chromosome 7) that is responsible for the polymorphism of P15 hindgut attachment (Fig. 4). Both phenotypic and genotypic evidence, thus, establish our prediction that alternative paths of infection can be associated with different host loci.

A broad peak spanning about 3 MB is visible in a close-up of contig 11 on chromosome 4 (Fig. 4b). An area about 400 kb wide within the peak region shows allele differentiation close to zero (called hereafter the gap region, Fig. 4, b and c). Mapping depth and SNP quality were similar to the flanking regions, but the number of SNPs was strongly reduced in the gap region, suggesting that the TN-RA-21 genotype is homozygote in the gap region (Supplementary Figs. 2–4).

### Refining the position of the G locus

**Fig. 5.**
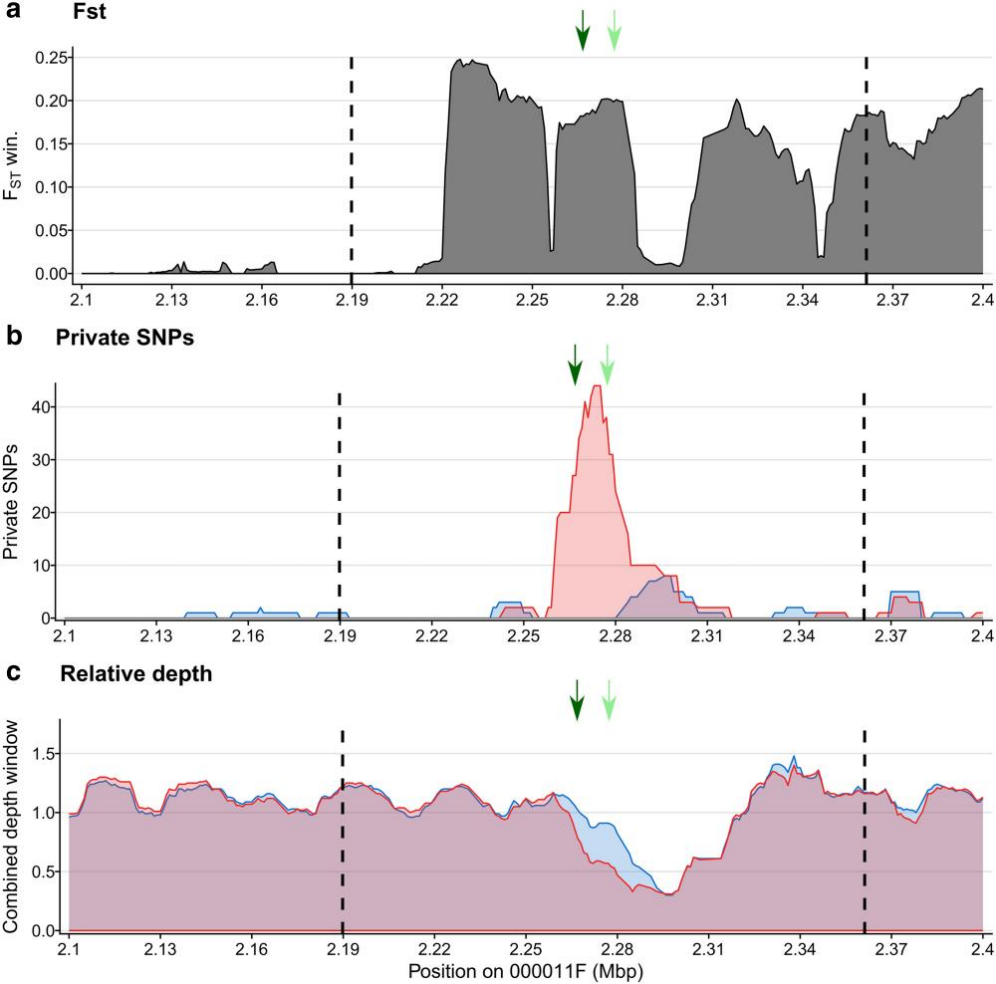
a) Fst, b) private alleles content, and c) coverage metrics in the resistant and susceptible pools (blue and red, respectively) along the region of the Pasteuria resistance Complex (PRC), a cluster of several *P. ramosa* resistance loci including the A,B,C and F loci in *D. magna*. The PRC is positioned within an hyperdivergent haplotype on contig 11 of chromosome 4 and is here delineated with dashed lines. Arrows indicate the position of the two candidate genes for the G locus (dark green for the *lactosylceramide 4-alpha-galactosyltransferase-like* gene and light green for the *putative Alpha–fucosyltransferase C* gene, respectively). Each graph is based on 10 kb windows with a 1 kb resolution.

Pool-sequencing data are often used to compute differentiation metrics (such as Fisher Exact Test or *F*_st_) that allow us to associate SNPs with phenotypic variation across different pools (Schlötterer *et al*. 2014). Although these differentiation metrics usually provide insight about the location of a specific locus, mapping accuracy is proportional to the number of recombination events that occurred between the two pools. In our case, because we used the self-offspring of one genotype, the number of recombination was limited, so indeed, the peak we detected on contig 11 of chromosome 4 was very broad (Fig. 4). To refine the position of the locus, thus, we developed a method using contrasting assumptions from the two hypothesized gene models for the G locus (Fig. 5), inspired by a technique that has been used to map sex determinant loci, which are typically single Mendelian loci with perfect dominance (e.g. Wen *et al*. 2022). Since the G locus is predicted to be a Mendelian segregating locus, we expected one pool to be homozygote at the G locus (genotype *gg*) and the other to be a mix of heterozygotes (*Gg*) and homozygote (*GG*). Using this reasoning, we computed private allele content at the contig 11 in each pool, where one pool should contain only the private G allele (the pool being *GG* and *Gg*), which should be absent in the other pool (*gg*). Private alleles were found in the susceptible pool (∼2260000–2290000 bp on the contig 11, Fig. 5), but not in the resistant pool, suggesting that the G locus is dominant for susceptibility, which supports genetic model 2. This fine mapping of the G locus revealed its position within the PRC (Fig. 5) in a window of about 30 kb. The same conclusion was reached using filters applied directly to allele frequencies to select SNPs for which one pool is homozygote (major allele frequency of one pool over 90%, see Supplementary Fig. 5): When keeping only homozygote variants in the susceptible pool, no other allele frequency differences were detectable. Thus, we are confident that the G locus is most likely a single locus, dominant for susceptibility (model 2 in Fig. 2).

The method we present here could be strengthened using coverage metrics, which also contain information about the heterozygosity of an individual at a genomic region of interest. However, the PRC is positioned within a large, highly variable and non-recombining region (HDH-4-1) (Naser-Khdour *et al*. 2024) that maps poorly to the reference genome. Coverage metrics were hence not able to reliably refine the G locus location in our case. Even so, it pointed at the same position inside the PRC. If the G locus had not fallen into a Hyper Divergent Haplotype (i.e. where significant structural variation occurs between genotypes), it is reasonable to assume that we would have been able to further refine its location window. We hence propose that applying private allele and coverage differences to pool-sequences can be an efficient way to fine map loci for plausible gene models.

Interestingly, our findings suggest that susceptibility is dominant in the G locus, which brings up a commonality regarding the genetic interactions of the C locus. Susceptibility is also dominant in the E locus, while for the A and B loci (as well as the C locus), resistance is dominant. The C locus nullifies the effect of the E and the G loci when a *D. magna* genotype carries two recessive alleles (*cc*) at the C locus, but the effects of the A and the B locus are canceled when an individual carries at least one dominant allele (*CC* or *Cc*) at the C locus. Hence, we can hypothesize that the genotype *cc* nullifies the effect of loci where susceptibility is dominant, while the genotype *C-* nullifies loci where resistance is dominant. This observation may shed light on the complex epistatic interactions of *Daphnia* resistance genes against *Pasteuria*. Our results notably strengthen the idea that epistatic interactions between Mendelian segregating loci are common principle in *D. magna* resistance mechanisms to various isolates of *P. ramosa* (Metzger *et al*. 2016; Ameline *et al*. 2021).

### Candidates genes for the G locus

Overall, we recorded 24 significant SNPs whose resistant pool was almost homozygote (major allele frequency over 90%) (Table 2, Supplementary Fig. 5). Our analysis contrasts a genome pool where we expect the recessive allele (*g*) to be fixed, with another pool where homozygotes (*GG*) and heterozygotes (*Gg*) are mixed. Assuming Hardy-Weinberg proportions, the frequency of the dominant *G* allele in the other pool should be around 66% (1/3 *GG* and 2/3 *Gg*); however, our data on the most significant SNPs suggest that its frequency is mostly slightly under 50% (range 30 to 50% for the private allele frequency in the susceptible pool; Table 2), making it slightly less prevalent than expected. This deviation from Hardy-Weinberg expectations might indicate that the *G* allele is at a disadvantage and may explain the overall low prevalence of P15F susceptible genotypes (see Supplementary Table 1) since they are all characterized by the presence of at least one *G* allele.

**Table 2.** Candidate SNPs significant for allele frequency change between the resistant and the susceptible pools and for being homozygotes in the resistant pool, the contig where they are positioned, their position within the contig, the two allele states with the private allele underlined, the private allele frequency in the susceptible pool (Private allele freq. S pool), the minimum coverage at the SNP position, the corrected *P*-value (i.e. False Discovery Rate, FDR) of the Fisher Exact Test, the annotation of the gene if the SNP fell into a gene, the gene region when relevant (intron or exon), the type of mutation caused by the SNP (S for synonymous and NS for non-synonymous) and the amino acid change for each state of the allele when the mutation is non-synonymous

Contig	position (bp)	Alleles	Private allele freq. S pool	Minimum coverage	FDR	Gene	Gene region	Mutation	Amino acid change
000011F	591,855	A/T	0.449	102	0.001	—	—	—	—
000011F	2,247,748	A/G	0.439	152	<0.001	Putative GMP synthetase [glutamine-hydrolyzing]	intron	—	—
000011F	2,262,428	G/T	0.463	175	<0.001	—	—	—	—
000011F	2,262,436	C/T	0.445	164	<0.001	—	—	—	—
000011F	2,264,118	A/G	0.495	184	<0.001	lactosylceramide 4-alpha-galactosyltransferase-like	exon	NS	His/Pro
000011F	2,264,119	T/G	0.5	182	<0.001	lactosylceramide 4-alpha-galactosyltransferase-like	exon	NS	His/Pro
000011F	2,265,835	C/A	0.328	128	0.004	lactosylceramide 4-alpha-galactosyltransferase-like	exon	S	—
000011F	2,265,848	A/G	0.344	122	0.002	lactosylceramide 4-alpha-galactosyltransferase-like	exon	NS	Val/Ala
000011F	2,265,856	C/T	0.336	122	0.008	lactosylceramide 4-alpha-galactosyltransferase-like	exon	S	—
000011F	2,266,035	C/T	0.395	145	<0.001	lactosylceramide 4-alpha-galactosyltransferase-like	intron	—	—
000011F	2,270,164	T/G	0.299	139	0.003	lactosylceramide 4-alpha-galactosyltransferase-like	exon	NS	Thr/Pro
000011F	2,270,180	C/T	0.327	136	0.001	lactosylceramide 4-alpha-galactosyltransferase-like	exon	S	—
000011F	2,277,106	A/G	0.432	173	<0.001	putative Alpha–fucosyltransferase C	exon	S	—
000011F	2,277,110	C/T	0.448	163	<0.001	putative Alpha–fucosyltransferase C	exon	NS	Ser/Tyr
000011F	2,277,111	T/A	0.444	171	<0.001	putative Alpha–fucosyltransferase C	exon	NS	Ser/Tyr
000011F	2,277,115	C/T	0.44	160	<0.001	putative Alpha–fucosyltransferase C	exon	NS	Lys/Glu
000011F	2,277,117	T/C	0.458	168	<0.001	putative Alpha–fucosyltransferase C	exon	NS	Lys/Glu
000011F	2,277,595	T/C	0.347	163	0.008	putative Alpha–fucosyltransferase C	exon	S	—
000011F	2,277,601	G/A	0.323	161	0.006	putative Alpha–fucosyltransferase C	exon	S	—
000011F	2,277,613	A/T	0.307	153	0.007	putative Alpha–fucosyltransferase C	exon	S	—
000011F	2,402,052	T/G	0.352	51	<0.001	hypothetical protein	exon	NS	Lys/Thr or Lys/Asn
000011F	2,443,847	C/T	0.609	110	<0.001	—	—	—	—
000011F	2,481,474	A/T	0.374	107	0.003	—	—	—	—
000011F	2,481,487	G/T	0.457	124	<0.001	—	—	—	—

The 24 significant SNPs fell into four different genes in the reference genome and caused non-synonymous mutations in exons in three of them (a *lactosylceramide 4-alpha-galactosyltransferase-like* (=LGT), a *putative Alpha–fucosyltransferase C* (=Fut), and a hypothetical protein, see Table 2). Most of the SNPs were concentrated in the glycosylation genes (LGT and Fut), with 8 SNPs falling into each of the two, making them strong candidates for the G locus. Some of these amino acid changes could impact protein properties, since in some cases, the alternative amino acid belongs to a different structure group than the reference amino acid. This is true for the lysine and the glutamic acids, for example, in the putative Fut sequence that belong to the basic and the acidic R group, respectively (Table 2).

The top two candidates for the G locus are relevant not only because of functional changes that might exist between resistant and susceptible genotypes to P15F attachment, but also for biological reasons. Galactosyltransferases and fucosyltransferases, are enzymes belonging to the Glycolsyltransferase family that assemble polysaccharide chains by either adding a sugar on a protein or a lipid, or by extending an existing saccharide chain (Almeida *et al*. 1997; De Vries *et al*. 2001; Ma *et al*. 2006; Li *et al*. 2018; Rini *et al*. 2022). These enzymes are promising candidates for resistance polymorphism, as some pathogens attach to sugars on the host epithelium before penetrating. Thus, specific sugars may be the frontline of the host's interaction with the pathogen (Audfray *et al*. 2013). Furthermore, glycosyltransferase enzymes are known to have specificity for donors and/or receptors, which would provide a mechanism for a matching-allele model of coevolution, such as *D. magna* and *P. ramosa* (Luijckx *et al*. 2013; Rini *et al*. 2022). Fucosyltransferases have already been suggested as a candidate for two other resistance loci against *P. ramosa* in *D. magna* (the C locus and the F locus, (Bento *et al*. 2017; Fredericksen *et al*. 2023)). On the pathogen side, it has been shown that collagen-like proteins such as Pcl7 in *P. ramosa* are likely involved in the attachment process (Huessy *et al*. 2024) and that the glycosylation of collagen-like protein in the parasite and/or of cuticle receptors of the host may explain the specificity of *P. ramosa* attachment patterns. A recent study further highlighted the high diversity in fucosyltransferase genes in *D. magna*, suggesting they might play an essential role (Naser-Khdour *et al*. 2024) in *P. ramosa* resistance. However, it is challenging to relate the top two candidate genes specifically to the G locus because it is within the PRC, which exists in a large non-recombinant region of the *D. magna* genome (Bento *et al*. 2017). The lack of homology between different haplotypes for this region poses mapping challenges that can impact our understanding of the gene's content among different *D. magna* haplotypes. Consequently, SNPs may be preferentially detected in more conserved regions of the PRC, potentially lacking functional association with a specific target locus. Alternatively, essential genes that confer resistance to pathogens undergo strong selection pressure to be maintained at the population level. The resistance genes in the PRC have been shown to be under long-term balancing selection (Luijckx *et al*. 2013; Bourgeois *et al*. 2021; Cornetti *et al*. 2024). We hence suggest that the *lactosylceramide 4-alpha-galactosyltransferase-like* gene and the *putative Alpha-fucosyltransferase C* gene we identified as candidate genes for the G locus are very likely to play a role in *P. ramosa* resistance. Nevertheless, based on our data, we cannot definitively determine whether one of our top candidate genes is the G locus or not. Future research that considers multiple *D. magna* genomes, such as a pangenome approach (Sherman and Salzberg 2020), rather than a single reference, might be necessary to advance our understanding of the genomic basis of *D. magna* resistance to *P. ramosa*.

## Supplementary Material

jkae302_Supplementary_Data

## Data Availability

The genomic data generated in this study have been deposited in the NCBI database under accession code BioProjectID PRJNA1143412. Scripts and data required for replicating our results are available at https://doi.org/10.6084/m9.figshare.25921390. Supplemental material available at G3 online.
