# Supplementary material for: Genetic basis of resistance in hosts facing alternative infection strategies by a virulent bacterial pathogen
Source: G3 (Bethesda). 2024 Dec 21;15(3):jkae302. doi: 10.1093/g3journal/jkae302 (PMC11917490; doi:10.1093/g3journal/jkae302)
Supplement: jkae302_Supplementary_Data [file jkae302_supplementary_data.zip › Supplemental_Material_Legends_G3-2024-405424.docx]

# Supplemental Material Legends

**Figure S1:** Manhattan plots representing the -log10(p-values) of the Fisher Exact Test conducted on alleles frequencies between the resistant and susceptible pools, plotted as a function of genomic position across the whole genome for each triplicate of TN-RA-21 offspring.

**Figure S2:** Read coverage calculated in 50 kb sliding windows and across contig 11 for a) the resistant pool and b) the susceptible pool. The black lines indicate the delineations of the gap region, while the orange lines indicate the position of the Pasteuria Resistance Complex.

**Figure S3:** SNP calling quality calculated in 500 SNPs sliding windows and across contig 11 for a) the resistant pool and b) the susceptible pool. The black lines indicate the delineations of the gap region, while the orange lines indicate the position of the Pasteuria Resistance Complex.

**Figure S4**: Number of SNPs called in 50kb sliding windows and across contig 11 for a) the resistant pool and b) the susceptible pool. The black lines indicate the delineations of the gap region, while the orange lines indicate the position of the Pasteuria Resistance Complex.

**Figure S5:** Manhattan plots representing the -log10(p-values) of the Fisher Exact Test conducted on allele frequencies between the resistant and susceptible pools, plotted as a function of genomic position across contig 11 for TN-RA-21 offspring depending on different filtering strategies based on allelic frequencies: a) Showing no filtering on the SNPs; b) SNPs filtered so their frequencies in the susceptible pools are over 90% (i.e., susceptible pool almost homozygote), and c) SNPs filtered so their frequencies in the resistant pools are over 90% (i.e., resistant pool almost homozygote). The orange lines indicate the coordinates of the Pasteuria Resistance Complex. SNPs in green are those retained for the investigation of the candidate gene for the G locus.

**Table S1:** Metrics generated by the co-occurrence test on attachment patterns between the *P. ramosa* isolate C1, C19 and P15 on a panel of 174 genotypes of *D. magna.* For each pair of *P. ramosa* isolates we show the number of attachments for the first isolate, the number of attachments for the second isolate, the number of observed co-occurrences (between *P. ramosa* strains), the number of expected co-occurrences, the probability of co-occurrence, the p-value associated with a negative co-occurrence (p-value -), the p-value associated with a positive co-occurrence (p-value +), the corrected p-value associated with a negative co-occurrence (FDR -), and the corrected p-value associated with a positive co-occurrence (FDR +). P-values were corrected using Benjamini & Hochberg false discovery rate (Benjamini and Hochberg 1995). Bold values refer to significant associations.

**Table S2:** Presentation of the 39 *Daphnia magna* genotypes used in the breeding experiment along with their resistotype for the attachment of *P. ramosa* isolates C1, C19, P15 hindgut (P15H) and P15 foregut (P15F). R stands for resistant and S for susceptible. The last two columns indicate if sexually produced F1 offspring were produced by selfing the genotype.

**Table S3:** Description of the samples sequenced in pool-sequencing, including the genotype of their parent, their resistotype for P15 foregut attachment, size of the pool and number of generated sequences.

**Table S4:** Percentage of observed and expected P15 foregut attachments (P15F) based on gene model 1 or gene model 2 for the G locus in the breeding experience. Genotypes that did not show segregation of resistance for P15 foregut attachment and are a) homozygous recessive at the C-locus (cc) and b) homozygous dominant (CC) or heterozygous (Cc) at the C-locus and homozygous at the G-locus (GG or gg depending on what model is considered). Genotypes that show segregation of resistance to P15 foregut attachment and are c) heterozygous (Cc) at the C-locus and homozygous recessive (gg) at the G-locus and d) homozygous dominant (CC) at the C-locus and heterozygous (Gg) at the G-locus. Some genotypes produced no selfed offspring but showed P15 foregut attachment (genotypes FI-GE-3-11, PL-KNP-P4, PL-W2-1, US-SP131-1, US-SP15-1, US-SP163-1, US-SP221-1 and US-SP6-13).
