# Supplementary figures and images for: Genetic basis of resistance in hosts facing alternative infection strategies by a virulent bacterial pathogen
Source: G3 (Bethesda). 2024 Dec 21;15(3):jkae302. doi: 10.1093/g3journal/jkae302 (PMC11917490; doi:10.1093/g3journal/jkae302)

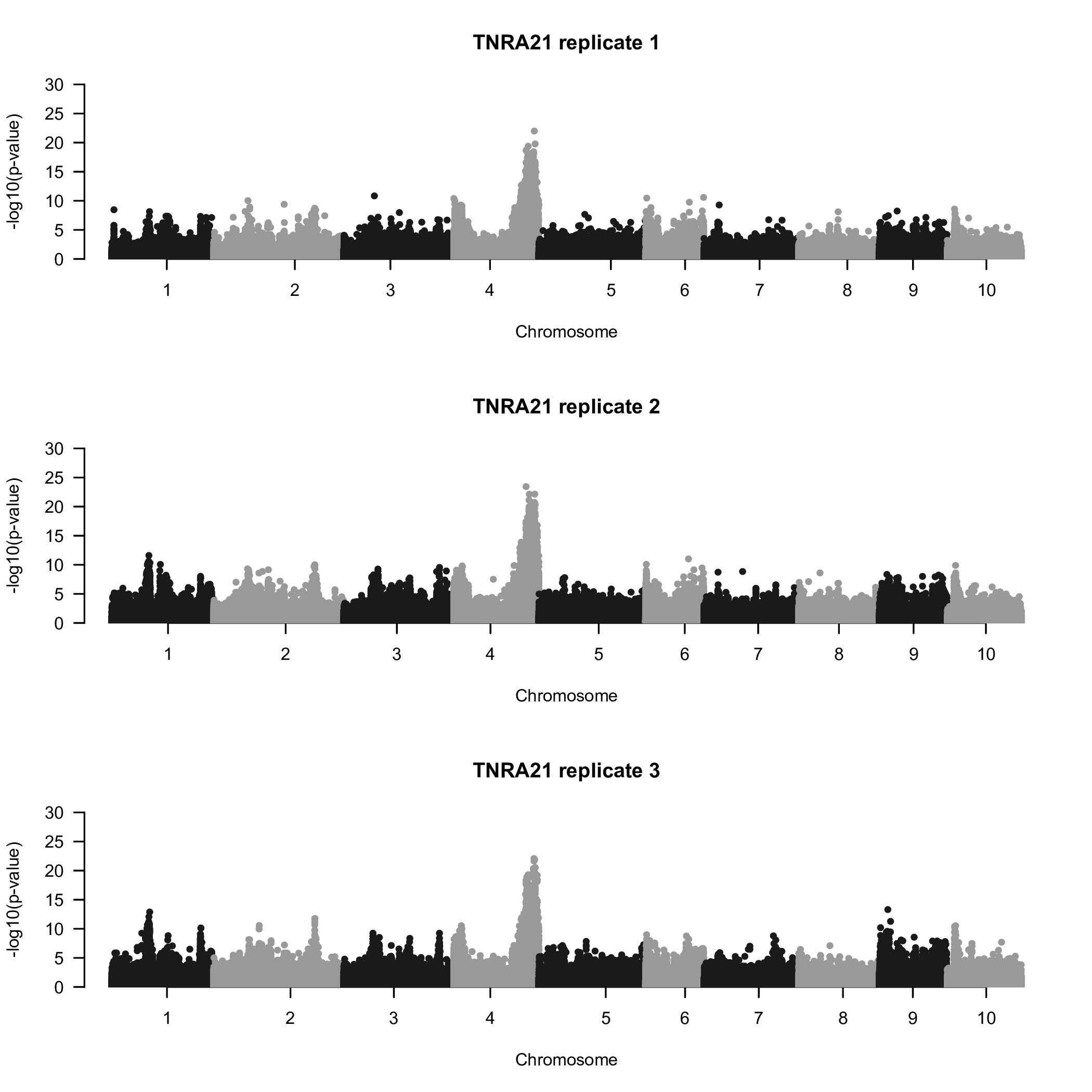

Supplement: jkae302_Supplementary_Data [file jkae302_supplementary_data.zip › Figure_S1_G3-2024-405424.tif]

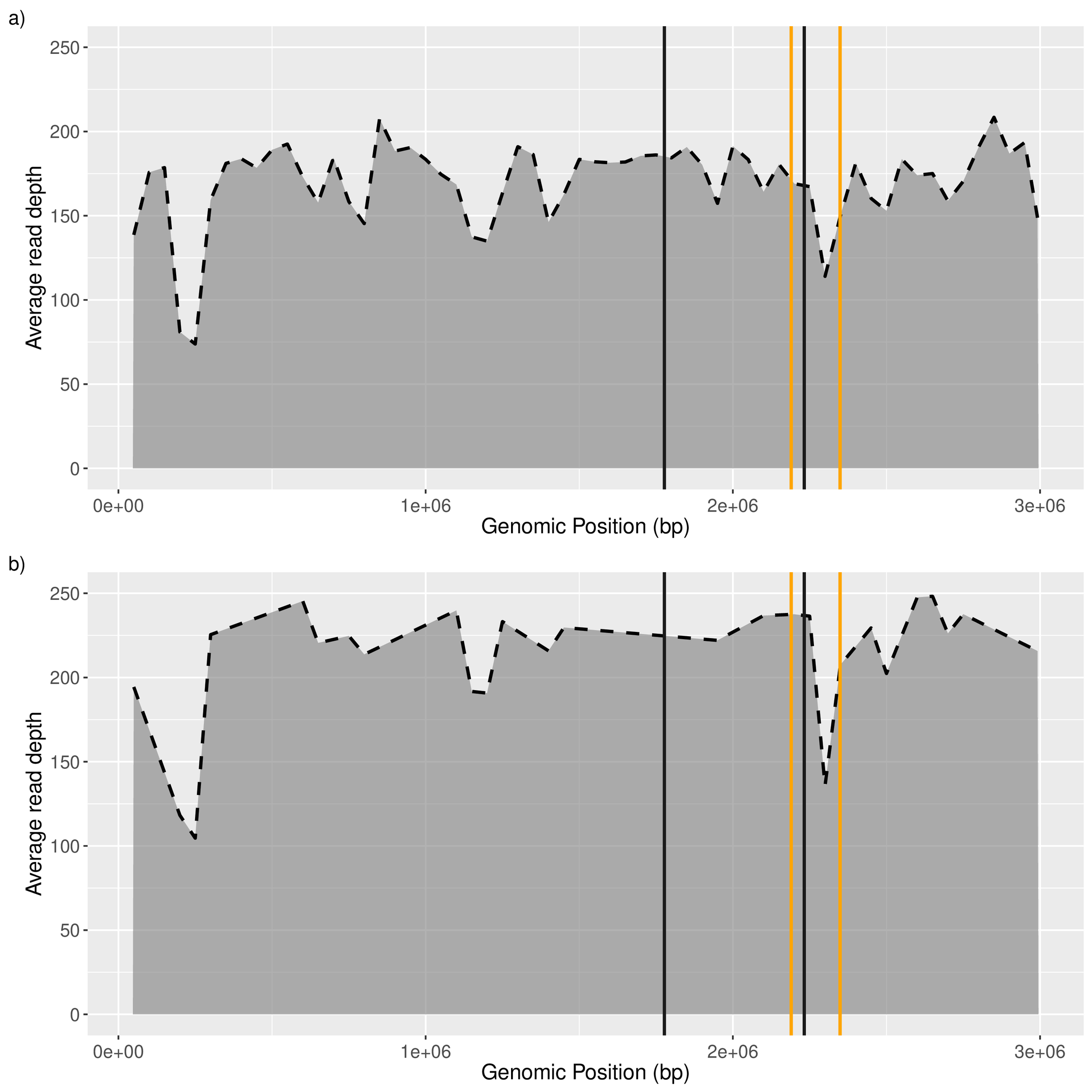

Supplement: jkae302_Supplementary_Data [file jkae302_supplementary_data.zip › Figure_S2_G3-2024-405424.tif]

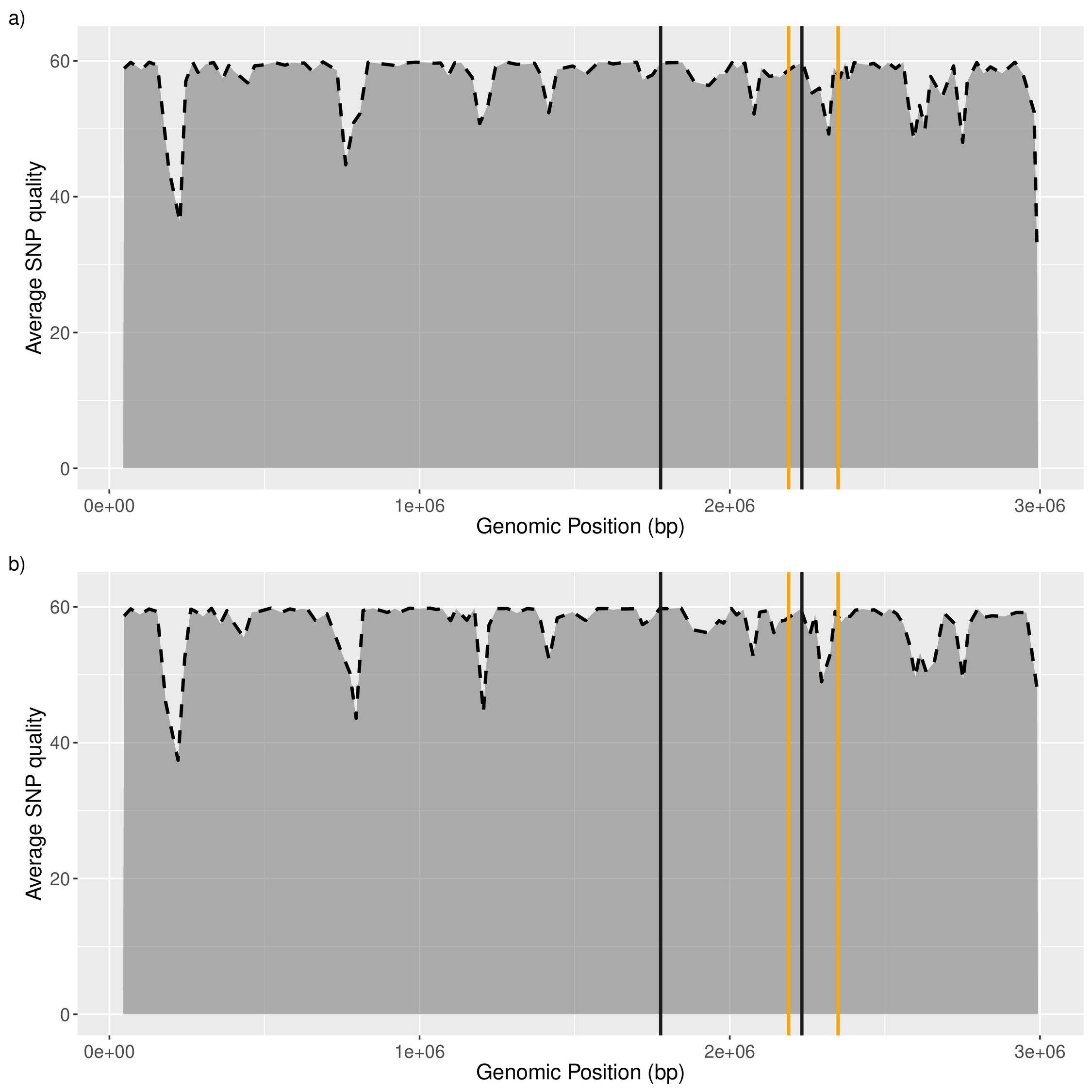

Supplement: jkae302_Supplementary_Data [file jkae302_supplementary_data.zip › Figure_S3_G3-2024-405424.tif]

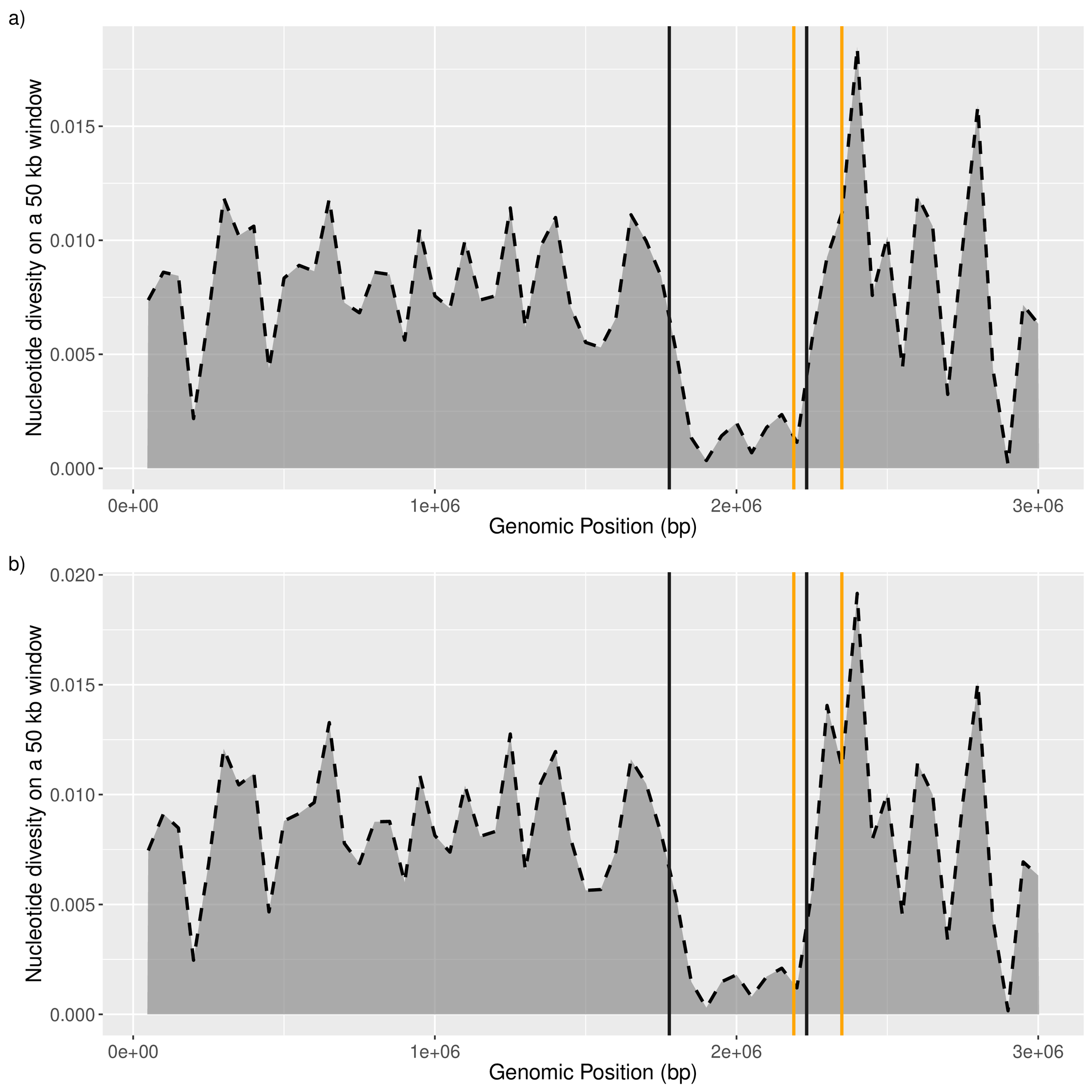

Supplement: jkae302_Supplementary_Data [file jkae302_supplementary_data.zip › Figure_S4_G3-2024-405424.tif]

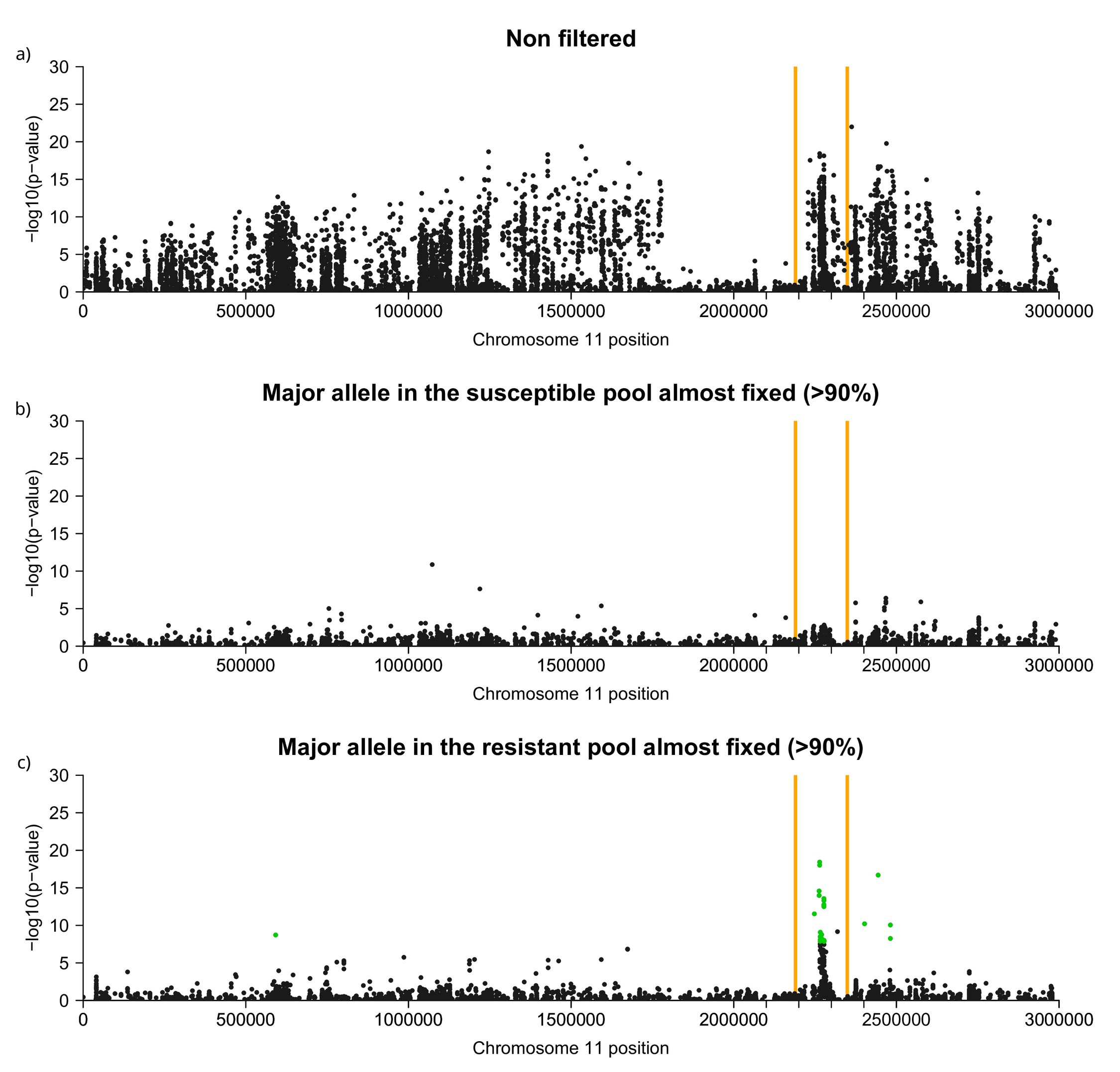

Supplement: jkae302_Supplementary_Data [file jkae302_supplementary_data.zip › Figure_S5_G3-2024-405424.tif]
